# The Prognostic Value of FoxP3+ Tumour-Infiltrating Lymphocytes in Rectal Cancer Depends on Immune Phenotypes Defined by CD8+ Cytotoxic T Cell Density

**DOI:** 10.3389/fimmu.2022.781222

**Published:** 2022-01-24

**Authors:** Sören Schnellhardt, Johannes Hirneth, Maike Büttner-Herold, Christoph Daniel, Marlen Haderlein, Arndt Hartmann, Rainer Fietkau, Luitpold Distel

**Affiliations:** ^1^ Department of Radiation Oncology, Universitätsklinikum Erlangen, Friedrich-Alexander-Universität Erlangen-Nürnberg, Erlangen, Germany; ^2^ Comprehensive Cancer Center Erlangen-Europäische Metropolregion Nürnberg (CCC ER-EMN), Erlangen, Germany; ^3^ Department of Nephropathology, Institute of Pathology, Universitätsklinikum Erlangen, Friedrich-Alexander-Universität Erlangen-Nürnberg, Erlangen, Germany; ^4^ Institute of Pathology, Universitätsklinikum Erlangen, Friedrich-Alexander-Universität Erlangen-Nürnberg, Erlangen, Germany

**Keywords:** Foxp3, CD8, regulatory T cells, rectal cancer, tumour-infiltrating lymphocytes, TILs, immune phenotypes, prognosis

## Abstract

Tumour-infiltrating FoxP3+ regulatory T cells have been identified as both positive and negative prognostic factors in colorectal cancer (CRC) and rectal cancer (RC). In this study we investigated whether immune phenotypes, defined by CD8+ cytotoxic T cell density, may influence the prognostic association of FoxP3+ T cell densities in RC. Tissue microarrays from 154 rectal cancer resections were immunohistochemically double stained for CD8 and FoxP3. CD8+ and FoxP3+ cell densities were measured in the stromal and intraepithelial compartment. Stromal FoxP3+ cell densities were not associated with 10-year overall survival (OS). In the “immune-desert” phenotype, defined by very low stromal CD8+ cell density, a high density of stromal FoxP3+ T cells displayed a tendency towards an association with decreased 10-year OS (p = 0.179). In “inflamed” tumours, defined by high intraepithelial CD8+ T cell infiltration, the opposite was the case and high stromal FoxP3+ T cell densities were a positive prognostic factor (p = 0.048). Additionally, patients with an increased FoxP3/CD8 cell density ratio demonstrated a strong trend towards decreased 10-year OS (p = 0.066). These contrasting findings suggest functional heterogeneity within the group of FoxP3+ T cells. They are consistent with experimental studies which reported suppressive and non-suppressive populations of FoxP3+ T cells in CRC. Furthermore, our study demonstrates that CD8 immunohistochemistry may act as an instrument to identify tumours infiltrated by possibly functionally differing FoxP3+ T cell subtypes.

## Introduction

The relevant relationship of immune cell infiltration in colorectal and rectal carcinomas (CRC) and patient prognosis has been established in numerous studies (1–4). Yet, our understanding of the complex causal links between immune response and disease progression remains limited. Various molecular subtypes have been identified and their immunological importance is becoming more apparent (5–7). A crucial role in influencing and controlling the local immune response is attributed to tumour-infiltrating FoxP3+ regulatory T cells (Treg) (8). Treg are an important element of immunological tolerance, both for self-tolerance in healthy tissue and locally in tumours (9, 10). Targeting this cell type is regarded as a promising approach for improving the response rate of current immunotherapies (11). However, in order not to cross the fine line between the induction of immunity in tumours and fatal systemic autoimmunity, a profound understanding of the role of Treg in cancer is necessary (12). But even the fundamental question of whether increased Treg infiltration in CRC is a favourable or unfavourable prognostic factor is still undecided and several studies have reported conflicting results (13, 14). In this immunohistochemical study we investigated the prognostic relevance of FoxP3+ T cell density in the context of different degrees of intratumoural inflammation as signified by CD8+ T cell infiltration. Our results offer a possible explanation for inconsistent findings on the role of Treg in previous studies and give insights into the intricacies of Treg functionality in cancers.

## Materials and Methods

### Patients and Samples

**Figure 1:**
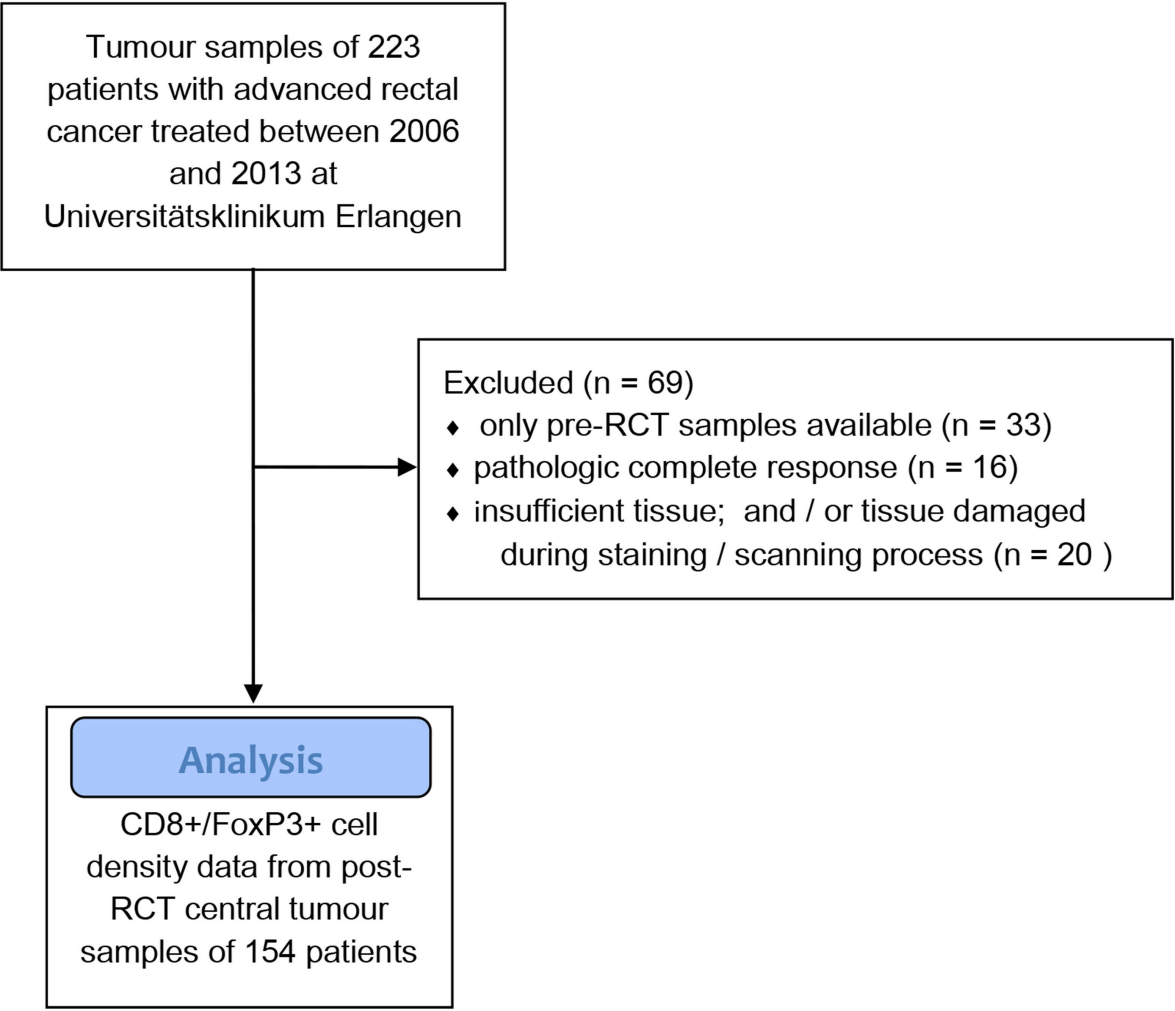
Flow diagram of the patient and tissue sample selection process.

Between 2006 and 2013, a total of 154 patients with advanced rectal cancer were treated at Universitätsklinikum Erlangen (University Hospital Erlangen) (**Table 1**). Tissue samples were obtained from surgical resection specimens after neoadjuvant treatment (**Figure 1**). The prognostic relevance of FoxP3+ and CD8+ T cell densities in general, cell-to-cell distances and distances to the epithelial-stromal interface have previously been reported in this cohort (15, 16).

**Table 1:** Clinical characteristics of the studied patient cohort.

Gender:	male: 118 (76.6%) female: 36 (23.4%)
Age (years):	mean: 63,8± 11,1; min.: 32 max.: 88
Primary tumour:	T1: 3 (1.9%) T2: 17 (11%) T3: 113 (73.4%) T4: 21 (13.6%)
Regional lymph nodes:	N0: 49 (31.8%) N1: 80 (51.9%) N2: 25 (16.2%)
Distant metastasis:	M0: 129 (83.8%) M1: 25 (16.2%)
UICC stage:	I: 11 (7.1%) II: 46 (29.9%) III: 72 (46.8%) IV: 25 (16.2%)
Grading:	G1: 3 (1.9%) G2: 121 (78.6%) G3: 30 (19.5%)
Chemotherapy:	5-FU: 48 (31.2%) 5-FU + Oxaliplatin: 89 (57.8%) other: 17 (11%)

The use of formalin-fixed paraffin-embedded material from the Archive of the Institute of Pathology was approved by the Ethics Committee of the Friedrich-Alexander-University of Erlangen-Nuremberg on 24 January 2005 (21_ 19 B), waiving the need for consent for using the existing archived material. Written informed consent was obtained “front door” from all patients allowing the collection of their tissue and clinical data.

### Treatment Protocol

All patients underwent neoadjuvant radiochemotherapy (RCT). A total radiation dose of 50.4 Gy was applied in 28 fractions of 1.8 Gy and 5-fluorouracil based chemotherapy as described earlier (17). After a period of 8 weeks treatment response was assessed and total mesorectal excision was performed to surgically remove the residual tumour. Additionally, 5-FU based adjuvant chemotherapy was administered.

### Tissue Microarray Construction and Immunohistochemistry

Two formalin-fixed, paraffin-embedded tissue cores of 2 mm diameter from the tumour centre of resection specimens of each patient were arranged in tissue microarrays (TMA). The tumour centre was identified and marked by a pathologist on a section of the whole tissue block prior to extraction of the cores. Samples of patients with pathologic complete response after neoadjuvant RCT were not included in this study.

An immunohistochemical double-staining with anti-FoxP3- and anti-CD8-specific antibodies was performed on the sections of the TMA to identify CD8+ and FoxP3+ T cells. As described previously, sections were deparaffinised and rehydrated and antigen retrieval was performed in a steam cooker for 5 minutes (16). The FoxP3-specific antibody (1:100, Ab20034, abcam, Cambridge, United Kingdom) was applied overnight and detected with a Polymer-Kit (Fa Zytomed POLAP-100) and Fast Red as a chromogen. Then, the CD8-specific antibody (1:50, M7103, Agilent, Santa Clara, CA United States) was added for 60 minutes and detection was performed with the Polymer-Kit and Fast Blue as a chromogen.

### Quantification of Cell Densities

Following immunohistochemical double-staining a high throughput scanner was used to scan sections at a magnification of 1:200 (Zeiss, Mirax MIDI Scan, Göttingen, Germany). Digital images of each tissue core were analysed using an image analysis software (Biomas, Erlangen, Germany). Stained cells were counted in a two-step process of automatic detection and manual correction by the examiner. Colour, shape, localization (nuclear for FoxP3 and membranous for CD8) and size were criteria for inclusion of stained cells. Cell densities (cells/mm²) were then calculated after measurement of the area of the stromal and intraepithelial compartment, respectively. For each patient the average cell density was determined by calculating the arithmetic mean of cell densities measured in the two cores.

### Statistical Analysis

Kaplan-Meier plots and the log-rank test were used to describe and compare overall survival rates (OS) and no-evidence-of-disease survival rates (NED) of different groups. Optimized cut-off values for prognostic groups were determined through receiver operating characteristic curve analysis and X-tile software (Version 3.6.1, Yale University, New Haven, Connecticut, USA) (18). Student’s t-Test and one-way analysis of variance (ANOVA) were used to compare means of two or more groups, respectively. Frequency distributions of categorical variables in contingency tables were compared with chi-squared test and Fisher’s exact test. SPSS (Version 26, IBM, Chicago, Illinois, USA) and Microsoft Excel (Version 16, Microsoft, Redmond, Washington, USA) were used to perform statistical analyses.

## Results

**Figure 2:**
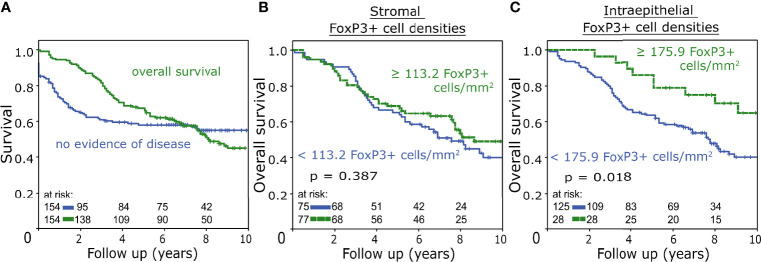
**(A)** Overall survival and no-evidence-of-disease survival rate of the studied patient cohort analysed with the Kaplan-Meier method and log-rank test. **(B, C)** Overall survival analysed with the Kaplan-Meier method and log-rank test according to FoxP3+ tumour-infiltrating T cell densities in the stromal **(B)** and intraepithelial **(C)** compartment.

Among 154 patients suffering from rectal cancer, the respective OS and NED were 45% and 60% at 10 years (**Figure 2A**). Overall stromal FoxP3+ cell densities were not associated with 10-year OS (**Figure 2B**). In the intraepithelial compartment high densities of FoxP3+ cells were associated with improved 10-year OS (p = 0.018) (**Figure 2C**).

**Figure 3:**
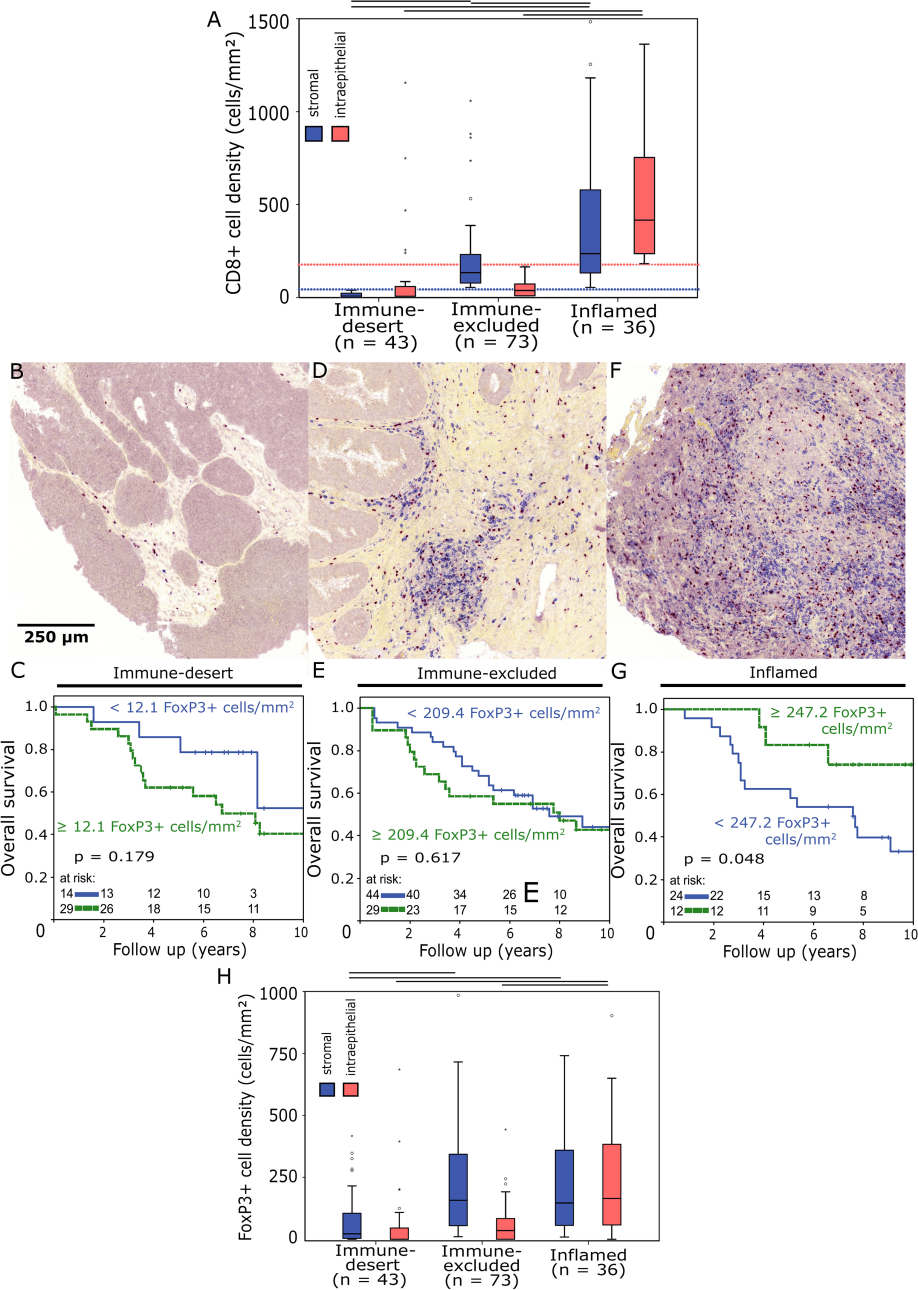
**(A)** Boxplots of stromal and intraepithelial CD8+ cell density distributions in different immune phenotypes. Outliers are marked with an asterisk (*). Horizontal black bars signify p < 0.05 in Student’s t-test. The “immune-desert” group was defined by stromal CD8+ cell densities < 40 cells/mm² (dotted blue line), “inflamed” tumours had intraepithelial CD8+ cell densities ≥ 170 cells/mm² (dotted red line). **(B, D, F)** Representative images of tissue microarray core sections from “immune-desert” **(B)**, “immune-excluded” **(D)** and “inflamed” **(F)** tumours double stained for FoxP3 (red)/CD8 (blue) (200x magnification). **(C, E, G)** Overall survival analysed with the Kaplan-Meier method and log-rank test according to stromal FoxP3+ tumour-infiltrating T cell densities in “immune-desert” **(C)**, “immune-excluded” **(E)** and “inflamed” **(G)** tumours. **(H)** Boxplots of stromal and intraepithelial FoxP3+ cell density distributions in different immune phenotypes.

Three groups defined by different “immune phenotype” were established based on stromal and intraepithelial CD8+ cell densities (**Figure 3A**). Cut-off values of these groups were defined by serial comparison of different CD8+ cell density thresholds for their impact on the prognostic significance of stromal FoxP3+ cell densities on OS and NED. Accordingly, “immune-desert” tumours were defined by stromal CD8+ cell densities of less than 40 cells/mm², which was accompanied by very low levels of intraepithelial CD8+ cell densities (**Figure 3B**). In this subgroup stromal FoxP3+ cell density displayed a tendency towards an association with decreased 10-year OS (52% vs 40% 10-year OS, p = 0.179) (**Figure 3C**). Tumours with stromal CD8+ cell densities of equal to or more than 40 cells/mm² in combination with intraepithelial CD8+ cell densities of less than 170 cells/mm² were assigned to the “immune-excluded” group, indicating a phenotype characterized by stromal accumulations of cytotoxic T cells with a simultaneous lack of intraepithelial infiltration (**Figure 3D**). 10-year OS was not associated with FoxP3+ cell densities in this group (**Figure 3E**). “Inflamed” tumours were defined by intraepithelial CD8+ cell densities of ≥170 cells/mm². With stromal and intraepithelial median values of CD8+ cell densities of 237 and 415 cells/mm², respectively, this group was characterized by high levels of cytotoxic T cell infiltration (**Figure 3F**). Here, increased stromal FoxP3+ cell densities were associated with significantly improved 10-year OS (33% vs. 74% 10-year OS, p = 0.048 (**Figure 3G**).

The prognostic value of intraepithelial FoxP3+ T cell densities also changed from negative to positive comparing “immune-desert” and “inflamed” tumours, but this relationship was less pronounced than in stromal FoxP3+ T cells (**Supplementary Figure 1**). FoxP3+ cell density levels correlated with those of CD8+ cells and were distinctly higher in “inflamed” tumours than in those classified as “immune-desert” (**Figure 3H**).

Stromal FoxP3+ and CD8+ cell densities were moderately positively correlated in both “immune-desert” (Spearman correlation coefficient = 0.59, p = 0.001, n = 43) and “inflamed” tumours (Spearman correlation coefficient = 0.407, p = 0.014, n = 36).

There were no significant differences concerning the type of chemotherapy used between immune phenotype groups (p = 0.067, **Supplementary Table 1**).

**Figure 4:**
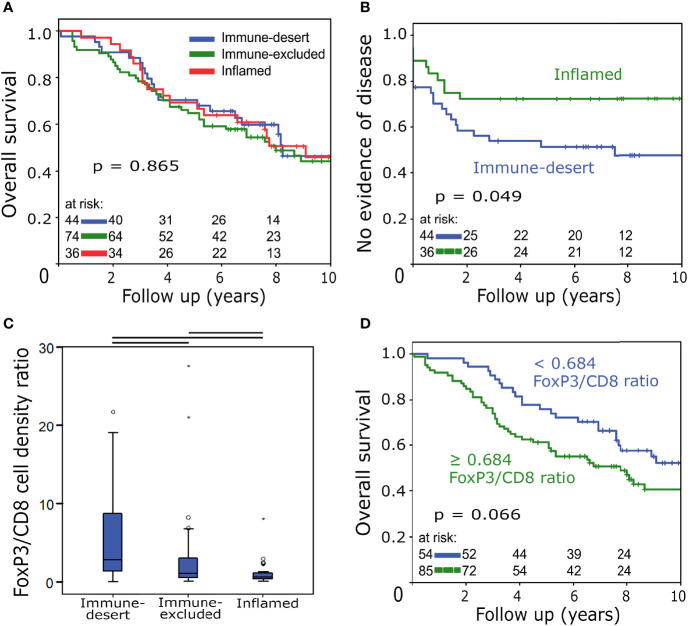
**(A)** Overall survival analysed with the Kaplan-Meier method and log-rank test according to immune phenotypes. **(B)** No evidence of disease status analysed with the Kaplan-Meier method and log-rank test according to immune phenotypes. **(C)** Boxplots of stromal FoxP3+/CD8+ cell density ratio distributions in different immune phenotypes. Horizontal black bars signify p < 0.05 in Student’s t-test. **(D)** Overall survival analysed with the Kaplan-Meier method and log-rank test according to the FoxP3+/CD8+ cell density ratio.

Comparing patients with different immune phenotypes, the 10-year OS was identical (**Figure 4A**). NED status on the other hand was significantly longer in the “inflamed” group compared to “immune-desert” (52% vs. 41% 10-year NED, p = 0.049) (**Figure 4B**). The only clinicopathological categories with significant differences between the analysed groups were age at diagnosis and nodal status: Patients with an “inflamed” tumour phenotype were older than those with “immune-excluded” and “immune-desert” tumours (median age at diagnosis: 69 vs. 67 vs. 60 years, respectively) and were more often lymph node positive at diagnosis (N+: 86% vs. 65% vs. 59%, respectively). The phenotype with the highest ratio of FoxP3+ to CD8+ cell densities was “immune-desert” (**Figure 4C**). An increased FoxP3/CD8 ratio was moderately associated with decreased 10-year OS (p = 0.066) (**Figure 4D**).

## Discussion

One of the most commonly used markers for self-tolerance promoting regulatory T cells (Treg) is the transcription factor forkhead box protein P3 (FoxP3) (19). The prognostic relevance of FoxP3+ tumour-infiltrating Treg in colorectal cancer (CRC) and rectal cancer (RC) has been a frequently studied albeit controversial subject. While Treg are generally assumed to have an immunosuppressive and thus adverse prognostic effect, there is ample evidence that high densities of intratumoural FoxP3+ T cells can be indicative of improved prognosis in CRC and other types of cancer (14, 20–22). There are, however, also numerous studies that reported the opposite, linking increased FoxP3+ T cell densities with an unfavourable prognosis (23–25). Some of these inconsistencies were most likely the result of variations in measurement methods and treatment modalities. But the apparent prognostic ambivalence is also indicative of the possibility that there may be multiple subpopulations of FoxP3+ T cells in CRC, or that Treg may have different and at times opposing functions depending on other environmental factors (10).

In the present study, we investigated the prognostic relevance of FoxP3+ T cell density in RC depending on the degree of inflammation within the tumour. To this end we established immune phenotypes defined by the density of tumour infiltrating CD8+ T cells. The phenotypes “immune-desert”, “immune-excluded” and “inflamed” have previously been described as archetypical immune constellations in tumours (26). Chen et al. explained tumoural immunological tolerance for each of these constellations with different underlying causes. “Immune-desert” tumours were defined as immunologically cold tumours attracting only very few inflammatory cells. Here, an immunosuppressive environment as well as a lack of antigens or antigen presentation were assumed to be main factors promoting immunological tolerance. One of several possible immunosuppressive elements in this context were FoxP3+ Tregs, which were thus regarded as an unfavourable prognostic factor in “immune-desert” tumours. In the “immune-excluded” phenotype, the cellular immune response was limited to surrounding stromal tissue only. Infiltration of the intraepithelial compartment by inflammatory cells did not occur. Consequently, lack of T cell migration into the tumour was presumed to be the tolerance promoting factor in this setting. Lastly, tumours with ubiquitous high densities of immune cells were designated to the “inflamed” group: Inflammatory cells were numerous but rendered ineffective by local factors like, for example, the PD-1/PD-L1 pathway. As suppression of a cellular immune response obviously failed in this setting, a different and possibly prognostically favourable function was assumed for FoxP3+ T cells.

In head and neck squamous cell carcinoma (HNSCC) we were able to use stromal and intraepithelial CD8+ T cell densities as surrogate markers for the above-defined phenotypes and could demonstrate that the prognostic relevance of FoxP3+ T cell density depended on the degree of intratumoural inflammation (27). The results of the present work confirm that this phenomenon can also be observed in RC. High overall stromal FoxP3+ T cell densities had no prognostic impact, whereas in the “immune-desert” and “inflamed” subgroups they correlated with decreased and improved survival, respectively. Similar observations were also made in other types of cancer: A large immunohistochemical study reported that high densities of FoxP3+ TIL were only indicative of improved survival in HER2+/estrogen receptor negative (ER-) breast cancer if they coincided with accumulations of CD8+ TIL. Simultaneously, the negative prognostic significance of FoxP3+ TIL densities in ER+ breast cancer depended on the absence of CD8+ T cells (28). Another parameter that underlines this relationship is the ratio of FoxP3+ and CD8+ cell density, which was significantly increased in “immune-desert” tumours compared to the remaining two phenotypes. An increased FoxP3/CD8 ratio was a predictor of reduced survival in this study as well as in cervical cancer, esophageal cancer and lung cancer (29–31). Although the calculation of a ratio is less complicated than the proposed classification into immune phenotypes, it does not adequately express the prognostic ambivalence of FoxP3+ TILs observed here. Nonetheless, all the previous reports suggest functional and prognostic heterogeneity within the group of FoxP3+ TILs, which could be an innate feature of this cell type not limited to specific cancer entities (11, 32).

Conclusions drawn from our cohort regarding the functional heterogeneity of FoxP3+ T cells in RC are limited by the size of the cohort and methodology as an immunohistochemical association study. One possible rationale for the observed results could be a suppressive function of FoxP3+ TILs in “immune-desert” tumours and a lack of function in “inflamed” tumours. Correlations between FoxP3+ and CD8+ TIL densities in the inflammatory phenotype could mean that the former are simply a concomitant phenomenon of cytotoxic T cell infiltration. An extensive work by Saito et al., however, provided a different and very reasonable explanation to distinguish FoxP3+ T cells in “immune-desert” tumours from those in “inflamed” tumours. They postulated that two types of activated FoxP3+CD4+ cells exist in CRC: suppression-competent Fraction II effector Tregs (Fr-II eTregs) and non-suppressive, pro-inflammatory Fr-III non-Treg FoxP3+ T cells (33). Based on the frequency of non-suppressive Fr-III FoxP3+ T cells, they subdivided CRC into type A and B tumours. Type A CRC were characterized by low numbers of Fr-III non-Treg FoxP3+ T cells and increased numbers of immunosuppressive genes expressed. Increased levels of FoxP3 transcripts in quantitative real-time PCR were associated with poor prognosis in this CRC subtype. This is consistent with our “immune-desert” phenotype, in which immunosuppression was reflected by very low densities of cytotoxic T cells and in which high FoxP3+ T cell densities were a negative prognostic factor. In type B CRC on the other hand, high FoxP3 expression was associated with an improved prognosis, an inflammatory environment prevailed and overall FoxP3 expression levels were described as significantly higher than in type A. The authors hypothesised that this could also be associated with an increased density of CD8+ cytotoxic T cells. Again, all this applies to our “inflamed” subtype: FoxP3+ T cell densities were significantly higher than in “immune-desert/type A-like” tumours and they were positively associated with survival. Additionally, highly increased densities of CD8+ cytotoxic T cells indicated a state of intratumoural inflammation. The lack of prognostic relevance of FoxP3+ T cells in the “immune-excluded” phenotype in our study can be explained in several ways. FoxP3+ T cells could be without prognostic relevance since, as stated earlier, lack of T cell migration into the tumour was assumed to be the main immune tolerance promoting factor in this phenotype. Alternatively, “immune-excluded” could also be an intermediate “type A/B” subtype in which neither Fr-II eTregs nor Fr-III non-Treg FoxP3+ cells predominate.

With regard to the prognostic relevance of the three immune phenotypes, the “inflamed/type B-like” group exhibited a significantly improved NED status in contrast to “immune-desert/type A-like”, which is again consistent with the results of Saito et al. The fact that this benefit does not translate to overall survival can be attributed to the fact that patients of the former group were considerably older and had a higher proportion of lymph node involvement.

Going forward we are planning to further characterise not only FoxP3+ Treg, but also CD8+ cytotoxic T cells, by evaluating the simultaneous expression of other important immunomodulatory factors in the phenotypes described here. The prognostically favourable “inflamed” phenotype with high Treg density could, for example, very likely contain a high percentage of CCR7+CD8+ T cells, which were described as a positive prognostic factor in CRC by Correale et al., especially in combination with increased Treg infiltration (34, 35).

In summary, our findings suggest that the prognostic relevance of FoxP3+ T cell density in RC depends on immune phenotypes defined by CD8+ cytotoxic T cell infiltration. In the context of our immunohistochemical study we can only speculate what distinguished FoxP3+ T cells in these individual subgroups. However, our findings are consistent with phenomena described by Saito et al. supporting the conclusion that there are suppressive and non-suppressive subpopulations of FoxP3+ T cells in CRC. The fact that it was possible to achieve very similar results with a completely different experimental approach underlines the possible role of two FoxP3+ T cell subtypes. These findings imply that the immunohistochemical detection of CD8+ T cells could serve as an inexpensive and widely available surrogate marker for identifying RC, which are predominated by either suppressive or non-suppressive FoxP3+ T cells. Clinically, this could become relevant for deciding whether a patient would benefit from a strategy of Treg depletion. Furthermore, other types of cancer like HNSCC or breast cancer should also be investigated more closely for functionally heterogenous FoxP3+ T cell subpopulations, as this feature and its prognostic relevance might not be limited to RC.

## Data Availability Statement

The raw data supporting the conclusions of this article will be made available by the authors, without undue reservation.

## Ethics Statement

The studies involving human participants were reviewed and approved by Ethics Committee of the Friedrich-Alexander-University of Erlangen-Nuremberg. The patients/participants provided their written informed consent to participate in this study.

## Author Contributions

Conceptualisation: LD, MB-H, CD, and SS. Resources: RF, AH, MB-H, CD, MH, and LD. Data Collection: SS, JH, and MH. Data Analysis & Interpretation: SS, LD, and JH. Writing - Original Draft: SS and LD. Writing - Review & Editing: LD, MB-H, CD, and SS. Supervision: LD, RF, MB-H, and CD. All authors contributed to the article and approved the submitted version.

## Conflict of Interest

The authors declare that the research was conducted in the absence of any commercial or financial relationships that could be construed as a potential conflict of interest.

## Publisher’s Note

All claims expressed in this article are solely those of the authors and do not necessarily represent those of their affiliated organizations, or those of the publisher, the editors and the reviewers. Any product that may be evaluated in this article, or claim that may be made by its manufacturer, is not guaranteed or endorsed by the publisher.
