# Supplementary material for: The Prognostic Value of FoxP3+ Tumour-Infiltrating Lymphocytes in Rectal Cancer Depends on Immune Phenotypes Defined by CD8+ Cytotoxic T Cell Density
Source: Front Immunol. 2022 Jan 24;13:781222. doi: 10.3389/fimmu.2022.781222 (PMC8818710; doi:10.3389/fimmu.2022.781222)
Supplement: Supplementary file 1 [file DataSheet_1.docx]

Supplementary Material

# Supplementary Figures


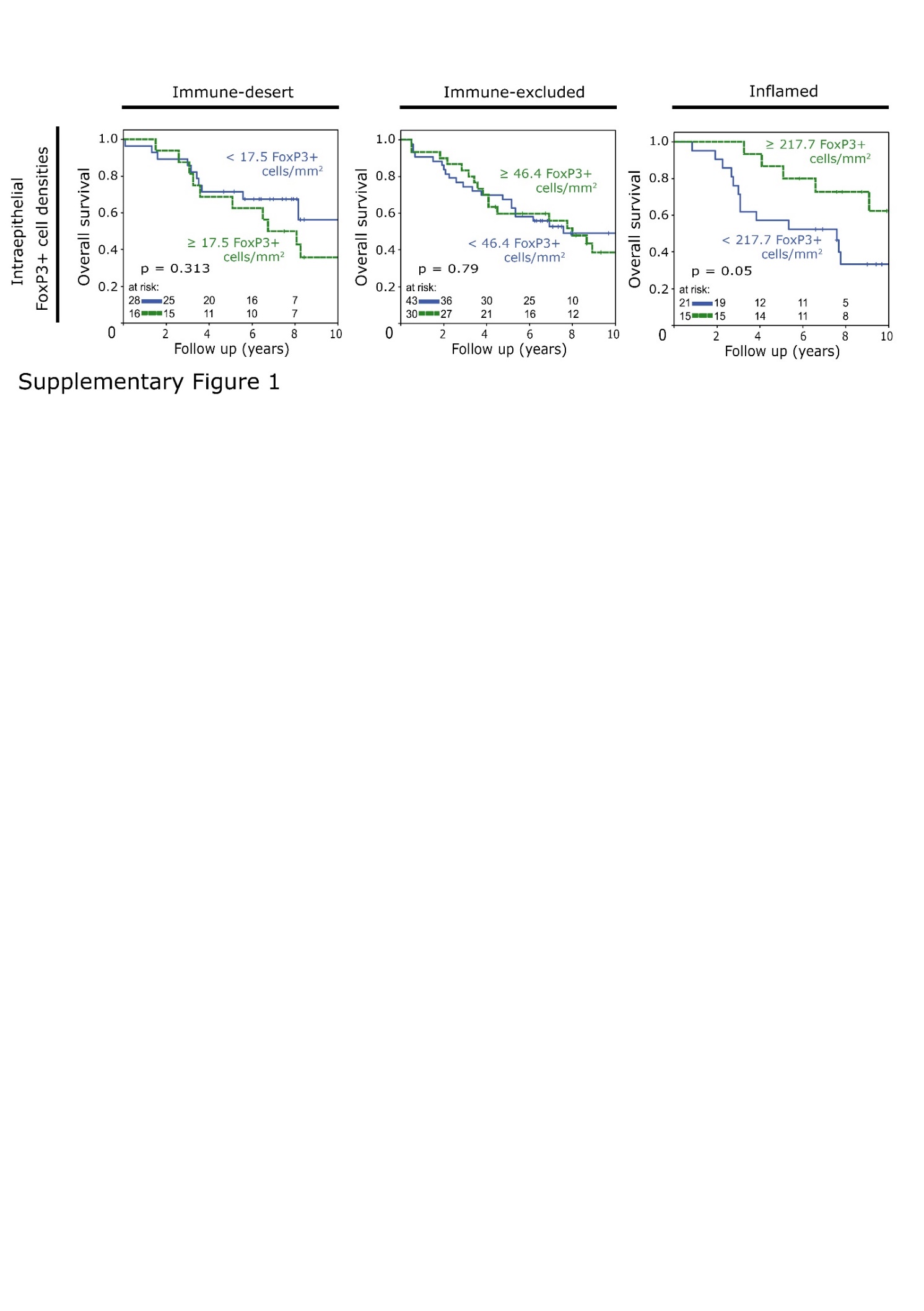


**Supplementary Figure 1.** Overall survival analysed with the Kaplan-Meier method and log-rank test according to intraepithelial FoxP3+ tumour-infiltrating T cell densities in “immune-desert”, “immune-excluded” and “inflamed” tumours.

# Supplementary Tables

**Supplementary Table 1.** Chemotherapeutic agent used according to immune-phenotypes.

| Chemotherapy: | | Immune phenotype | | |
| --- | --- | --- | --- | --- |
|  |  | Immune-desert | Immune-excluded | Inflamed |
| 5-FU | n (% of column) | 10 (22.7) | 26 (35.6) | 12 (33.3) |
| 5-FU + Oxaliplatin | n (% of column) | 25 (56.8) | 45 (60.8) | 19 (52.8) |
| other | n (% of column) | 9 (20.5) | 3 (4.1) | 5 (13.9) |
